# Hypermucoviscosity in *Klebsiella pneumoniae*: manifestation, regulation, and roles in pathogenesis

**DOI:** 10.1128/jb.00123-26

**Published:** 2026-07-27

**Authors:** Tamal Dey, Manoharan Shankar

**Affiliations:** 1 Microbial Physiology Laboratory, Department of Bioscience and Bioengineering, Indian Institute of Technology Jodhpur 231955, Jodhpur, Rajasthan, India; University of Southern California, Los Angeles, California, USA

**Keywords:** pathogenesis, regulation, hypervirulence, hypermucoviscosity, *Klebsiella pneumoniae*

## Abstract

In recent years, *Klebsiella pneumoniae* (Kpn) has evolved into a global public health threat. Convergent Kpn lineages that are both hypervirulent and multidrug-resistant have emerged and can cause community-acquired, drug-resistant infections, with increased mortality. A major driver of hypervirulence in Kpn is hypermucoviscosity (HMV), a phenotype historically associated with increased capsular polysaccharide (CPS) production. Recent findings show that HMV is a distinct trait characterized by increased CPS chain length and uniformity, rather than by overproduction of CPS. The regulators of mucoid phenotype (RmpADC and/or RmpA2) have traditionally been characterized as determinants of HMV. However, recent evidence suggests that Kpn can also manifest HMV independently of the regulators of mucoid phenotype, driven by missense mutations in the *wzc* gene of the CPS biosynthesis locus. These findings have immense ramifications, as non-hypervirulent Kpn lineages can rapidly enhance their virulence and invasiveness by acquiring *wzc* mutations and manifesting HMV. Since HMV confers Kpn with the ability to adhere poorly to immune cells and resist phagocytosis, it enables systemic dissemination. This benefit, however, appears to be niche specific, as HMV hinders adhesion to epithelial cells and biofilm formation on medical devices, traits which may be beneficial in some niches. Kpn tackles this problem by fine-tuning HMV expression via switches driven by mutations or through regulatory control in response to environmental cues. This review synthesizes recent findings on the molecular mechanisms by which HMV is manifested, is intricately regulated, and how HMV contributes to Kpn’s success in diverse infection niches, such as the respiratory tract, bloodstream, and urinary tract.

## INTRODUCTION

*Klebsiella pneumoniae* (Kpn) is a gram-negative, rod-shaped, non-motile bacterium that was first isolated by Carl Friedlander from the lungs of patients who had succumbed to pneumonia in 1882. Kpn is a facultative anaerobe found in various environments, including water, soil, and the human gut. Kpn is an opportunistic pathogen, a member of the ESKAPE group of pathogens, often causing hospital-associated infections such as ventilator-associated pneumonia, urinary tract infections (UTIs), bacteremia, wound infections, etc. (1). In the late 1980s, more severe, invasive infections, mostly manifesting as pyogenic liver abscesses caused by Kpn, were reported from Taiwan, often in community settings. Usually occurring in individuals with diabetes or otherwise healthy individuals, the Kpn isolates causing these infections could effectively spread within the host and disseminate to distal body sites, causing secondary infections such as endophthalmitis and meningitis (2, 3). Although such “hypervirulent” Kpn isolates were first reported from Taiwan and other Southeast Asian countries, similar reports emerged later from Europe and North America (4–7).

Based on disease severity, Kpn can broadly be classified into classical strains, which infect immunocompromised patients in hospital settings, or hypervirulent strains, which can cause severe infections in apparently healthy individuals in community settings. Classical strains are usually multidrug-resistant, carrying resistance genes on plasmids or on other mobile genetic elements (8). On the other hand, hypervirulent strains carry hypervirulence factors on large virulence plasmids and mobile genetic elements, but are mostly susceptible to antibiotics (8, 9). As expected, with great concern, classical lineages have acquired hypervirulence-conferring virulence determinants, and hypervirulent lineages have acquired multidrug resistance genes, resulting in convergent lineages that can cause severe, community-acquired infections and cannot be treated with conventionally used and last-resort antibiotics. Several studies worldwide have confirmed the emergence of such untreatable, convergent lineages, which are a serious threat to global public health (8, 10–14).

One of the defining features of hypervirulent Kpn isolates is a virulence-associated phenotype called hypermucoviscosity (HMV) (5, 15–18). Kpn strains produce a characteristic capsular polysaccharide (CPS), which envelops the cell and serves as a critical, defensive virulence factor. By modifying the physical properties of its CPS layer, often subject to genetic regulation and environmental modulation, Kpn can become hypermucoviscous. A “string test” is commonly used to test for HMV in bacteria. The formation of a viscous string >5 mm in length when bacterial colonies on an agar plate are stretched with an inoculation loop is considered a string test-positive result (15). Since the string test has an accuracy of 90% in predicting hypervirulence in Kpn (19), the terms hypermucoviscous and hypervirulent became synonymous, although this may not always be the case (20). For example, the carriage of genes involved in the biosynthesis of the siderophores salmochelin (*iro* locus), aerobactin (*iuc* locus), and other uncharacterized genes (*peg-344*) was also associated with a hazard ratio of >25 for severe disease/death in a murine sepsis model (19). Thus, HMV, along with other contributing virulence factors, is a hallmark of hypervirulent Kpn.

Given the importance of HMV in the virulence of Kpn, several recent studies have unraveled fascinating molecular mechanisms underlying HMV. Some of these studies also show that Kpn can modulate HMV manifestation via intricate regulatory networks that respond to both environmental cues and the infection niche. The ability to acquire, lose, and regulate HMV also enables Kpn to rapidly adapt to the distinct niches (respiratory tract, urinary tract, bloodstream, wound/tissue, etc.) it may encounter within the host. This work primarily aims to synthesize recent findings describing the various molecular mechanisms by which HMV can manifest and how HMV can be modulated via regulatory control or environmental cues. Furthermore, this review discusses how modulation of HMV contributes to hypervirulence and the ability of Kpn to infect distinct niches.

## MANIFESTATION OF HYPERMUCOVISCOSITY

### The regulators of mucoid phenotype: *rmpADC*/*rmpA2*

The first genetic locus associated with HMV was *rmpA* (the regulator of mucoid phenotype A), identified in 1989 on a virulence plasmid in a K2 serotype strain of Kpn (21). The study showed that a 1.6 kb segment of the virulence plasmid, when introduced into a non-mucoid strain of Kpn or *E. coli* HB101, conferred the HMV phenotype. Inactivation of the *rmpA* gene in the K2 Kpn strain led to a 1,000-fold virulence attenuation in a murine intraperitoneal (IP) infection model (21), highlighting the importance of the *rmp* locus for virulence. The same group showed that the mucoid phenotype observed upon introduction of *rmpA* into *E. coli* HB101 was due to enhanced colanic acid production as a result of RmpA exhibiting an RcsA-like activity (22). Shortly after the discovery of the *rmpA* locus in 1993, a variant was reported in Japan and annotated as *rmpA2* (23). The *rmpA2* gene was found on a large plasmid in Kpn “chedid,” a highly virulent strain belonging to serotype K2 that manifests HMV, and was reported to enhance CPS biosynthesis in this strain. The *rmpA2* coding sequence (636 bp) was slightly longer than that of *rmpA* (410 bp), leading to a ~70 amino acid longer RmpA2 protein. At the nucleotide level, however, *rmpA* and *rmpA2* share ~80% homology. A similar copy of *rmpA2* was also later discovered on the pLVPK virulence plasmid of Kpn CG43 (24). It may be noted, however, that despite its association with virulence plasmids, few studies demonstrate that *rmpA2* is functional. These findings established the notion in the field that carriage of the *rmpA/rmpA2* locus can confer HMV in Kpn. This was then believed to be due to elevated CPS biosynthesis, which enhances Kpn’s virulence and enables it to cause invasive disease.

In 2019, the notion that overproduction of CPS causes HMV was challenged. Walker and colleagues (25) identified another gene in ATCC 43816 KPPR1S, adjacent to the *rmpA* gene, which they called *rmpC*. The Δ*rmpC* mutant produced significantly lower levels of CPS than the parent, as measured by the uronic acid assay. Intriguingly, the Δ*rmpC* mutant remained HMV positive, as verified by a string test. Subsequently, RmpC was shown to transcriptionally activate CPS biosynthesis, and yet, its loss had no effect on HMV in ATCC 43816 KPPR1S. The authors also demonstrated that hypermucoviscosity could be quantified by sedimentation. The ratio between the OD_600_ of a Kpn suspension, measured before and after low-speed centrifugation, can be obtained and serves as an index of HMV. Called the sedimentation assay, this method can easily and reliably distinguish between HMV Kpn, which sediments poorly (a higher ratio), and non-HMV Kpn, which sediments well (a lower ratio). The Δ*rmpC* mutant exhibited an HMV phenotype comparable to that of the wild type despite having significantly lower levels of CPS. Thus, this study, for the first time, demonstrated that a strain can remain HMV-positive even when CPS production diminishes, thereby uncoupling elevated CPS levels from HMV (25).

**Fig 1:**
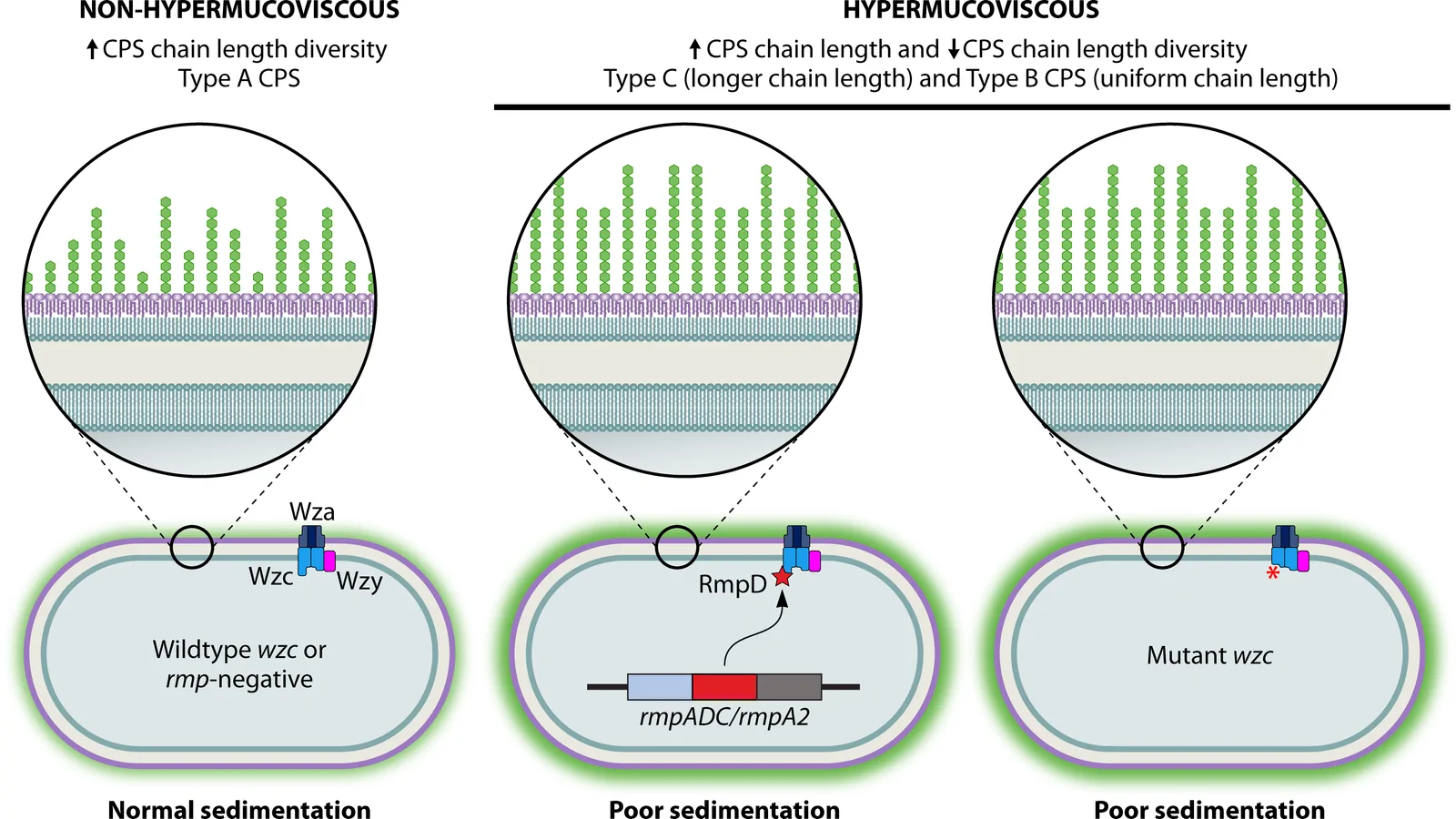
Molecular mechanisms of hypermucoviscosity (HMV) driven by the *rmpADC/rmpA2* loci and *wzc* mutations. At the molecular level, HMV can be achieved through two independent pathways. In the *rmp*-mediated pathway, *rmpA* induces the expression of *rmpD*. The resulting RmpD protein then interacts with the *Klebsiella pneumoniae* (Kpn) capsule synthesis machinery, specifically the tyrosine kinase Wzc, which regulates capsular polysaccharide (CPS) chain length. This interaction changes the CPS with diverse chain lengths (Type A) seen in *rmpD*-negative strains to longer-chain-length CPS (Type C), and uniform-chain-length CPS (Type B), which together drive the HMV phenotype. Alternatively, in *wzc* mutation-mediated HMV, specific mutations within the *wzc* gene directly cause Kpn to produce CPS with longer (Type C) and more uniform chains (Type B).

In 2020, the same group identified another short gene, *rmpD*, located between the *rmpA* and *rmpC* genes (26). Inactivation of *rmpD* resulted in the loss of HMV, as confirmed by a string test and sedimentation assay. Compared to the wild-type strain, the Δ*rmpD* mutant showed significantly reduced HMV but produced wild-type-level CPS. Thus, this study identified *rmpD* as the genetic factor underlying HMV. *rmpADC* was henceforth redefined as a three-gene operon, with RmpA acting as an autoregulatory activator. Therefore, these two seminal studies (25, 26) combined changed the notion that the manifestation of HMV requires elevated CPS production. However, it was not clear at the time how RmpD led to the hypermucoviscosity phenotype, although it was known that inactivating CPS biosynthesis, even in the presence of functional *rmpD,* leads to the loss of HMV. This finding is in agreement with an earlier study that employed transposon mutagenesis to identify the gene responsible for HMV in a pyogenic liver abscess isolate, NTUH-K2044 (15). This screen identified a 1.2 kb gene, the disruption of which led to a loss of HMV, enhanced susceptibility to complement-mediated killing, and virulence attenuation in a murine IP infection challenge. This gene was named *magA* (mucoviscosity-associated gene A) and was detected in 52 out of the 53 invasive strains characterized in the study. However, it was later found that *magA* encodes the capsule unit polymerase Wzy and is part of the Kpn K1 CPS biosynthetic locus (27–29). This suggested that the loss of HMV observed upon loss of *magA*/*wzy* is due to the inability to produce CPS. Furthermore, deleting the CPS biosynthesis locus genes or the positive regulators of the CPS locus genes (*rcsA*, *rcsB*, *kvrA*, *kvrB*, *kvg/kvh*, and *iscR*) can reduce CPS production and, consequently, reduce or abolish HMV (25, 30–33). Taken together, it is clear that a basal level of CPS biosynthesis is essential for the HMV phenotype, suggesting that HMV could result from CPS plus an unknown factor (25, 26). This was also considered plausible, as an earlier study had shown that chemical modification of the CPS with sialic acid enhanced the HMV phenotype in a K1 serotype HMV-positive strain. The removal of sialic acid with neuraminidase reduced the HMV of the neuraminidase-treated bacteria (34). Later, Mike and colleagues (31) conducted a systematic study to assess HMV and CPS biosynthesis using a transposon mutagenesis approach. This study identified mutants that were HMV^low^/CPS^low^, HMV^WT^/CPS^low^, HMV^low^/CPS^WT^, HMV^high^/CPS^WT^, HMV^WT^/CPS^high^, HMV^low^/CPS^high^, and HMV^high^/CPS^high^, suggesting that although HMV and CPS production are coordinated, they are dissociable. Later, Ovchinnikova and colleagues (35) conclusively demonstrated in a seminal study that the HMV polysaccharide is chemically no different from the CPS polysaccharide, as determined by nuclear magnetic resonance spectroscopy. Instead, they demonstrated that in Kpn KPPR1S and *E. coli* K30 carrying functional *rmpD*, the CPS chain length was increased and was more uniform. This means that some of the CPS chains were longer, and others had more or less similar numbers of CPS subunits per chain, making the chain lengths in HMV strains more uniform. Specifically, in *rmpD*-bearing strains, the CPS chain length was highly consistent, producing a modal cluster of CPS chains as observed by capsule SDS-PAGE. In sharp contrast, in the absence of *rmpD*, the CPS lacked uniformity in chain length, and the modal cluster is not visible on capsule SDS-PAGE. These findings suggested that the enhanced length and more uniform chain length of the CPS, rather than chemical modification of the CPS, led to HMV manifestation (Fig. 1). The authors suspected that such a regulation of the CPS chain length may be achieved by RmpD modulating the activity of one of the proteins involved in CPS biosynthesis and export.

The Kpn CPS is synthesized from oligosaccharide subunits usually comprising 3–6 sugars (often mannose, galactose, rhamnose, glucose, and fucose). The sugar monomers may be linked by different glycosidic linkages, may also contain branching side chains, and may be modified (e.g., pyruvylation, O-acetylation, or O-formylation). Furthermore, the incorporation of uronic acids (glucuronic acid and/or galacturonic acid) also imparts a negative charge to the CPS, which confers protection from host immune defenses (36). The CPS is typed either by serotyping (K-type) or biosynthesis locus typing (KL-type). Given the complex structure and various modifiable components in the CPS, it is not surprising that there is enormous diversity in the CPS serotypes and KL-types (37), although some K-types, such as K1 and K2, have been associated with higher virulence (38). Kpn CPS biosynthesis is known to operate via the Wzy-dependent pathway. The process begins with an initial glycosyltransferase (WcaJ/WbaP) in the cytoplasm, which transfers a sugar substrate to the undecaprenyl phosphate lipid carrier, inserted into the membrane. Additional glycosyltransferases encoded within the variable region of the CPS biosynthesis locus add serotype-specific sugar molecules to synthesize the CPS repeat unit. This repeat unit is then flipped across the inner membrane by the Wzx flippase. Then, the Wzy polymerase is believed to polymerize the repeat units. Wzy, being a distributive enzyme, releases the product after every repeat unit is added. Thus, the Wzc copolymerase is necessary to regulate the polymerization, chain length, and export of the synthesized CPS (39). Very recently, Yuan and colleagues (40) presented an interesting model for Wzc-mediated regulation of CPS polymerization and export. This hypothetical model is based on (i) recent structural studies (41) on the WzyE polymerase and WzzE copolymerase complex involved in the synthesis and export of the enterobacterial common antigen and (ii) an AlphaFold3-predicted structure of the *E. coli* CPS polymerase Wzy and copolymerase Wzc. The authors suggest that dephosphorylation of Wzc by Wzb likely promotes the formation of an octameric complex surrounding a single molecule of Wzy, which together form the polymerization platform. The jelly roll domain of Wzc binds the CPS subunit, which is flipped across the inner membrane by Wzx, and delivers it to Wzy. As the CPS polymer grows, Wzc aligns its helical arm with the assembled Wza translocon. The mature CPS is then translocated via the Wza translocon across the outer membrane and is believed to be anchored to the cell surface by Wzi. Wzc then autophosphorylates, leading to the dissociation of the polymerization platform, allowing the system to restart (40). Using bacterial two-hybrid analysis, Ovchinnikova and colleagues (35) demonstrated that RmpD could interact with Wzc (tyrosine autokinase), Wzx (flippase), and Wzy (capsule unit polymerase). The strongest signal, however, was observed for Wzc, the tyrosine autokinase encoded within the capsule biosynthesis locus. Taken together, these important findings support the current understanding in the field that RmpD may bind Wzc and, through an unknown mechanism, modulate its activity to enhance the length and uniformity of the CPS chains (35) (Fig. 1).

Given the well-characterized role of the *rmpADC* and *rmpA2* loci in HMV and their association with hypervirulence, it is important to understand the prevalence of these loci and their sequence variants from a population genomics perspective. The *rmpADC* locus was detected in 992/13,993 (7.08%) Kpn species complex genomes (42). In our recent analysis of a collection of 22,569 Kpn genomes from over 80 countries, *rmpADC* was detected in 1,358/22,569 genomes (6.01%), while *rmpA2* was detected in 1,796/22,569 genomes (7.95%) (37). The *rmp* loci can be carried either chromosomally, on plasmids, or on other mobile genetic elements, and some strains are known to carry multiple *rmp* loci. For example, in Kpn NTUH-K2044, which is a serotype K1 pyogenic liver abscess isolate, a large virulence plasmid carries one copy each of *rmpA* and *rmpA2* loci, while a chromosomally encoded *rmpA* locus was also found. Lam and colleagues (42) classified *rmpADC* loci into five lineages (*rmp1*, *rmp2*, *rmp2A*, *rmp3*, and *rmp4*) on the basis of their sequence. Among these, *rmp1* (KpVp-1) and *rmp2* (KpVp-2) were carried mostly on plasmids, while *rmp2A* could be carried chromosomally or on plasmids. *rmp3* and *rmp4* were mostly carried chromosomally, with *rmp3* being mostly carried on an ICE*Kp1* element. More importantly, representative loci from all sequence variants could enhance CPS biosynthesis and also confer HMV in non-HMV Kpn isolates (43).

Since their discovery and association with hypervirulent Kpn isolates, *rmpADC* and *rmpA2* have garnered the most interest as the genetic determinants of HMV. However, as early as 2006, several reports demonstrating HMV in strains of the Kpn species complex lacking *rmp* loci have emerged (44–51). Our group also reported Kpn P34, an unusually hypermucoviscous strain isolated from pus recovered from a hepatic, solid, cystic gallbladder lesion in a patient. Hypermucoviscosity was confirmed by both the string test and sedimentation assay. However, Kpn P34 does not overproduce CPS and is susceptible to complement-mediated killing. Genome sequencing confirmed that Kpn P34 does not carry any plasmids and that it does not harbor *rmpADC*/*rmpA2* loci on its chromosome (52). Such findings indicate that HMV in Kpn can manifest through mechanisms independent of *rmpADC*/*rmpA2* loci.

### HMV can manifest independent of *rmpADC/rmpA2* via *wzc* mutations

Several studies have reported that HMV can manifest independently of *rmpADC*/*rmpA2* in members of the *Klebsiella pneumoniae* species complex and in *E. coli* (Table 1). However, the mechanism behind *rmpADC*/*rmpA2*-independent HMV was not known until recently. Ernst and colleagues (53) identified both hypomucoid and HMV strains of Kpn in a collection of 54 ST258 isolates. It is noteworthy that ST258 isolates are considered “classical” Kpn, which do not carry hypervirulence genes, including the *rmp* loci. Two ST258 HMV strains were identified from bloodstream infections and lacked both *rmpADC* and *rmpA2*. The authors screened for mutations in capsule regulator genes and found none but identified missense mutations in the *wzc* gene, located within the CPS locus of Kpn.

**TABLE 1:** Members of the *Klebsiella pneumoniae* species complex and *E. coli*, where hypermucoviscosity has been reported in the absence of *rmpADC/rmpA2* loci

Sl. no	Species	*rmpADC*/*rmpA2* absence confirmed by	String test	Sedimentation	K type	Reference
1	*Klebsiella quasipneumoniae*	Genome sequencing	Unknown	Unknown	Unknown	(54)
2	*Klebsiella quasipneumoniae*	Genome sequencing	Positive	Unknown	Unknown	(55)
3	*Klebsiella pneumoniae*	PCR	Positive	Unknown	Unknown	(45)
4	*E. coli*	Genome sequencing	Positive	Unknown	N/A^*a*^	(56)
5	*Klebsiella pneumoniae*	Genome sequencing	Positive	Unknown	KL54	(46)
6	*Klebsiella pneumoniae*	Genome sequencing	Positive	Unknown	K108	(48)
7	*Klebsiella pneumoniae*	Genome sequencing	Positive	Unknown	KL19	(50)
8	*Klebsiella variicola*	Genome sequencing	Positive	Poor	KL114	(47)
9	*Klebsiella variicola*	Genome sequencing	Positive	Unknown	KL25	(50)
10	*Klebsiella pneumoniae*	Genome sequencing	Positive	Unknown	K2	(51)
11	*Klebsiella variicola*	Genome sequencing	Positive	Unknown	KL30	(57)
12	*E. coli*	Genome sequencing	Positive	Unknown	N/A	(58)
13	*Klebsiella pneumoniae*	Genome sequencing	Positive	Poor	KL131	(52)

^*a*^ N/A, not applicable.

The Wzc protein, a bacterial tyrosine kinase (BY-kinase), is encoded within the CPS biosynthetic locus in Kpn and in the group 1 capsule locus of *E. coli*. In *E. coli*, it is believed to regulate the polymerization and export of the CPS (39). The *E. coli* homolog of Wzc, which has been characterized best and is highly homologous to Kpn Wzc, is an inner membrane protein with periplasmic and cytoplasmic regions. The cytoplasmic C-terminal region not only includes the BY-kinase domain, including the Walker A, Walker A′, and Walker B motifs, but also contains a tyrosine-rich tail, where Wzc is believed to be poly-phosphorylated. Recent studies on *E. coli* Wzc have revealed that dephosphorylated Wzc is octameric, while multi-phosphorylated Wzc is monomeric (59, 60). The CPS biosynthesis locus in Kpn and *E. coli* group 1 capsule loci also encodes the Wzb phosphatase, which is necessary for the dephosphorylation of Wzc. The authors suggest that cycling of Wzc between the multi-phosphorylated (monomeric) and dephosphorylated (octameric) states may be critical for regulating CPS production, CPS chain length, and its export.

In the study by Ernst and colleagues (53), missense mutations in *wzc* resulting in the substitutions G565A/S/R or the substitution A535E within the C-terminal kinase domain of Wzc could confer hypercapsule and HMV. The authors go on to show that isolates bearing *wzc* mutations could emerge *in vivo* during UTIs in human patients; in a murine model of UTI, where non-HMV classical isolates were inoculated into mouse bladders; and also spontaneously during *in vitro* culture. Similarly, in the ST11 (sublineage 258, classical) clinical isolate GZ-1, the substitution S682N in Wzc resulted in hypermucoviscosity (61). Mutations in *wzc* have been shown to emerge in capsulated Kpn strains evolved in different structured environments (LB medium, artificial urine medium, artificial sputum medium, and soil medium), outside the host, leading to enhanced HMV in strains lacking *rmpADC*/*rmpA2* (62). In 2023, Khadka and colleagues (63) also identified *wzc* mutations leading to HMV in an *rmpD*-positive strain KPPR1S under conditions that suppress *rmpD*-mediated HMV. In-trans overexpression of the mutant Wzc alleles (G569C, G569V, and G569D) in non-HMV UTI isolates *Klebsiella variicola* Kv-616 and Kpn Kp-714 led to enhanced HMV and poor sedimentation. These findings are not uncommon, and several groups have reported diverse *wzc* mutations in the members of the Kpn species complex leading to HMV (Table 2). Such Wzc substitutions, which confer HMV, may not be without fitness trade-offs. A recent study showed that in a carbapenem-resistant, HMV strain CRB113, a Y572D substitution in Wzc was sufficient to confer HMV (64). However, the mutant was rendered more susceptible to complement-mediated killing.

**TABLE 2:** Variants of Wzc that have been reported to cause hypermucoviscosity in members of the *Klebsiella pneumoniae* species complex and their corresponding phenotypes

Location within Wzc	Wzc variant	Type	Strain	Emergence	Phenotype(s)	Reference
Periplasmic	L74P	Non-synonymous	Kpn UR_11	Emerged *in vivo* in a patient with UTI	Hypercapsule, HMV, poorly phagocytosed, enhanced dissemination, highly lethal in murine IP, UTI, and BSI infection models.	(53)
	Q395K	Non-synonymous		Spontaneous	HMV and sedimentation resistant	(63)
Cytoplasmic kinase domain	526InsA	–^*a*^		Spontaneous	HMV and sedimentation resistant	(63)
	G534E	Non-synonymous	Kva 342	Experimental evolution in artificial sputum medium	String-test positive	(62)
	A535E	Non-synonymous	Kpn UCI_38	Emerged after bladder inoculation of WT Kpn in toll-like receptor 4-deficient mice	HMV and poorly phagocytosed	(53)
	G540E	Non-synonymous	Kva 342	Experimental evolution in LB medium	String-test positive	(62)
	G540R	Non-synonymous	Kva 342	Experimental evolution in LB medium	String-test positive	(62)
	F557S	Non-synonymous	Kpn 318Kp	*In vivo* evolution after ceftazidime/avibactam treatment	Biliary abscess isolate	(65)
	G565A	Synonymous	Kpn UCI_38	Spontaneous emergence in *in vitro* culture	Hypercapsulated, poorly phagocytosed, enhanced dissemination, and enhanced mortality in zebrafish BSI model	(53)
	G565S	Non-synonymous	Kpn BIDMC_13 & BIDMC16	Emerged *in vivo* in clinical isolates	Hypercapsulated, HMV, and enhanced mortality in zebrafish BSI model	(53)
	G565R	Non-synonymous	Kpn UCI_38	Bladder inoculation of WT Kpn in toll-like receptor 4-deficient mice	Hypercapsule and poorly phagocytosed	(53)
	D567E	Synonymous	Kpn K2	*In vitro* evolution in the presence of ceftazidime/avibactam resistance	String-test positive	(66)
	G569C	Non-synonymous	KPPR1	Spontaneous	HMV and sedimentation resistant	(63)
	G569V	Synonymous	KPPR1	Spontaneous	HMV and sedimentation resistant	(63)
	G569D	Non-synonymous	KPPR1	Spontaneous	HMV and sedimentation resistant	(63)
	G596V	Non-synonymous	Kva 342	Experimental evolution in LB medium	String-test positive	(62)
	T569P	Synonymous	Kpn K4	*In vitro* evolution in the presence of ceftazidime/avibactam	String-test positive	(66)
	Y572D	Non-synonymous	CRB113	Emerged in a clinical isolate	HMV and sedimentation resistant	(64)
	D643H	Non-synonymous	Kva 342	Experimental evolution in artificial sputum medium	String-test positive	(62)
	D643E	Non-synonymous	Kva 342	Experimental evolution in artificial sputum medium	String-test positive	(62)
	P644L	Non-synonymous	Kpn K1	*In vitro* evolution in the presence of ceftazidime/avibactam	String-test positive	(66)
	P645Q	Non-synonymous	Kva 342	Experimental evolution in artificial urine medium	String-test positive	(62)
	P646Q	Non-synonymous	Kva 342	Experimental evolution in artificial sputum medium	String-test positive	(62)
	P647L	Non-synonymous	Kpn K3	*In vitro* evolution in the presence of ceftazidime/avibactam	String-test positive	(66)
	P646S	Synonymous	KPPR1	Spontaneous	HMV and sedimentation resistant	(63)
Close to the tyrosine-rich C-terminus	S682N	Synonymous	Kpn GZ-1	Emerged *in vivo* in a clinical isolate	HMV and decreased biofilm formation	(61)

^*a*^ –, not applicable.

Intriguingly, reports indicate higher frequencies of HMV emergence during treatment with ceftazidime-avibactam, via *wzc* mutations, both *in vivo* (65) and *in vitro* (66). The F557S substitution in Wzc emerged after a bloodstream infection caused by an ST512 (sublineage ST258, classical) Kpn strain was treated with ceftazidime-avibactam. The HMV isolate bearing this substitution in Wzc was recovered from a pericholecystic liver abscess in the same patient. Similarly, the prevalence of HMV among ceftazidime-avibactam-sensitive Kpn isolates (5.6%) was significantly lower than that in ceftazidime-avibactam-resistant isolates (46.7%). Experimental evolution of resistance to ceftazidime-avibactam *in vitro* also led to the development of HMV in around 40% of the resistant isolates (66). This phenomenon can perhaps be explained by antibiotic-stress-induced mutagenesis, as recently demonstrated in *Pseudomonas aeruginosa* (67). Stress-induced mutagenesis upon exposure to ceftazidime and avibactam, both *in vivo* and *in vitro*, has been shown to result in approximately threefold higher mutation frequencies, leading to the emergence of resistant and cross-resistant variants. Such enhanced mutation rates, combined with the need to evade immune cells within the host, could therefore lead to the selection of HMV Kpn strains carrying *wzc* mutations *in vivo*. Thus, stress-induced mutagenesis can not only drive the emergence of antibiotic resistance but also cause the emergence of HMV Kpn.

## REGULATORY AND ENVIRONMENTAL MODULATION OF HMV IN NICHE-SPECIFIC INFECTION PROGRAMS OF Kpn

Kpn can cause infection in different niches within the host, including the respiratory tract, urinary tract, bloodstream, liver, gall bladder, and spleen. These niches differ significantly in terms of the presence/absence of physical barriers, the types of immune cells present, and the nutrients they provide. Thus, to mount a successful infection across different niches, Kpn needs to adopt niche-specific infection programs, which are defined as the coordinated, spatiotemporally regulated expression of its critical virulence factors (CPS; lipopolysaccharides; HMV; siderophores: enterobactin, salmochelin, yersiniabactin, and aerobactin; type three fimbriae; and type one fimbriae) that determine success in each niche (68, 69). This is because the expression of a specific set of virulence factors and the repression of others may dictate success in a given environment. For example, in the bloodstream, enhanced production of CPS of certain K-types can create a physical barrier that prevents exposure of cell-surface antigens and hinders assembly of the membrane attack complex proximal to the outer membrane, thereby conferring immune evasion and resistance to complement-mediated killing (70–74). On the other hand, in the urinary tract, a thick capsule may be disadvantageous, as it may prevent adhesion to epithelial cells (53), occlude cell-surface adhesins (75), and interfere with biofilm formation (76). Along similar lines, it is important to understand the benefits and/or disadvantages conferred by HMV during infection in diverse niches.

**Fig 2:**
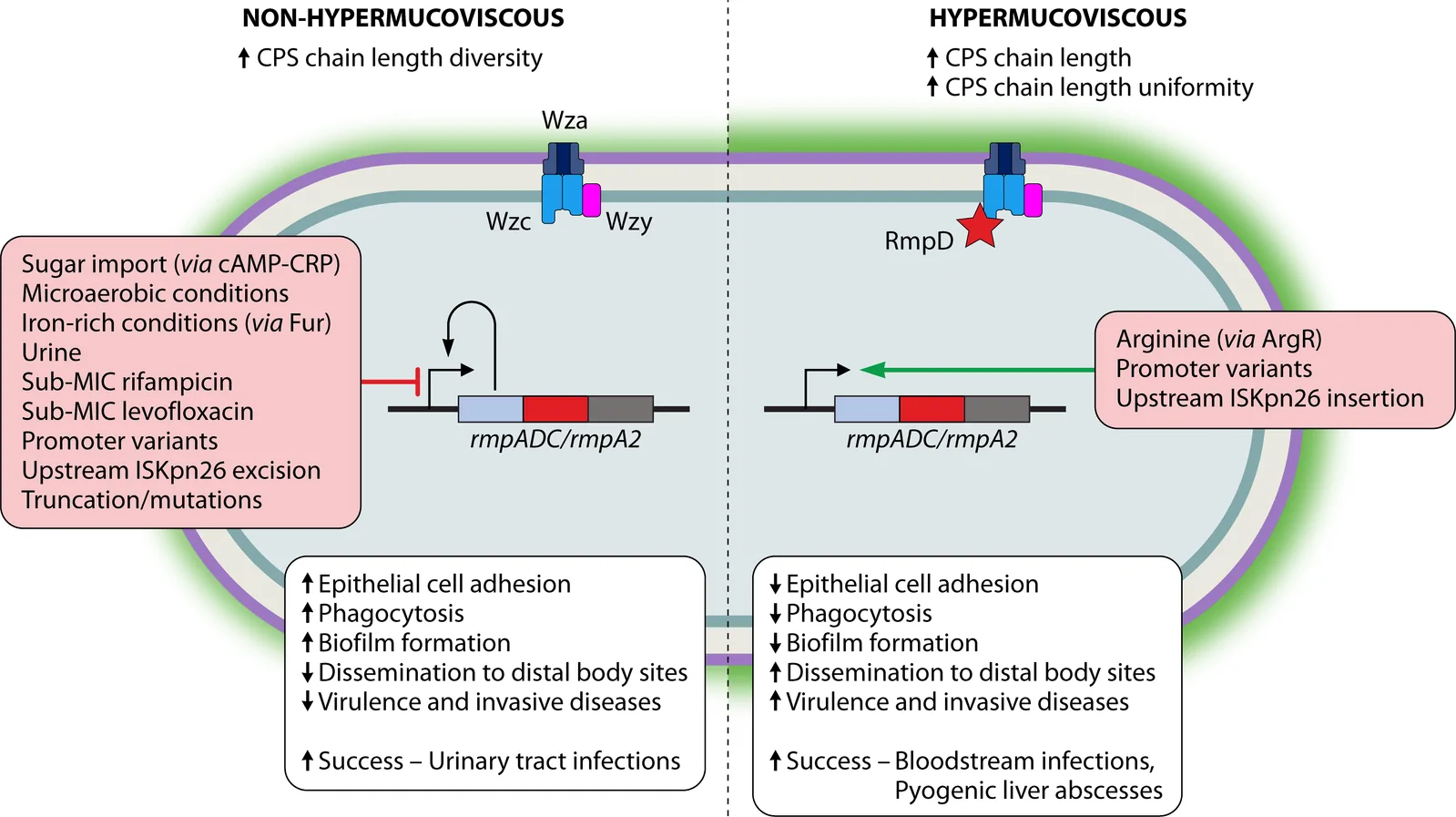
Niche-specific adaptation of *rmpD*-positive Kpn involves a transition between hypermucoviscous (HMV) and non-hypermucoviscous states, driven by diverse environmental and genetic factors. Furthermore, specific factors can activate HMV by modulating the expression of the *rmp* locus. For instance, the transcription factor ArgR, specific mutations in the *rmpA* promoter sequence, or the presence of the IS*Kpn*26 insertion sequence upstream of the *rmpA* gene have all been shown to upregulate HMV. On the other hand, exposure to urine, microaerobic environments, and iron-rich conditions seems to repress *rmpADC*-mediated HMV. Exposure to sub-inhibitory concentrations of certain antibiotics, such as rifampicin and levofloxacin, can also suppress the phenotype. Genetically, the excision of ISKpn26 from the *rmpA* promoter or the truncation and deletion of its upstream region leads to decreased HMV manifestation. Biologically, HMV provides a distinct advantage in environments like the bloodstream or in pyogenic liver abscesses. By reducing attachment to epithelial and immune cells, HMV minimizes phagocytosis, facilitating dissemination to distal body parts and increasing the Kpn invasive disease-causing ability. However, in specific infection niches, such as the urinary tract, HMV can be counterproductive. Successful colonization of the bladder requires strong attachment to epithelial cells; therefore, Kpn downregulates HMV in these environments to enhance adherence and biofilm-forming capacity, thereby successfully establishing infection.

Some HMV-negative Kpn isolates bearing mutated/truncated versions of *rmpADC*/*rmpA2*, leading to the production of truncated or inactive proteins, have been reported, suggesting that some environments can select for non-HMV populations even in *rmpADC*/*rmpA2*-positive Kpn strains (Fig. 2). A poly(G) tract in the *rmpA/rmpA2* coding sequence appears to be a hotspot for insertions and deletions, which change the length of the poly(G) tract, often resulting in truncated or inactivated proteins (42, 77). The length of the poly(G) tract can also fluctuate within a population, making it a form of phase variation (78, 79). This raises the possibility that in some niches, where *rmpADC*/*rmpA2*-mediated HMV could be disadvantageous, truncated/inactivated versions of the *rmp* loci may help alter Kpn’s infection program to better suit the niche. The inactivation of *rmpA* abolishes HMV and also decreases CPS biosynthesis (25). This observation can be explained by (i) the positive autoregulation of the *rmpADC* operon by RmpA, (ii) the finding that RmpC positively regulates the CPS biosynthesis locus, while not affecting HMV (25), and (iii) the discovery that RmpD is necessary for HMV but does not affect CPS biosynthesis (26). These findings also helped clearly delineate the benefits conferred by the CPS and HMV in the infection programs of Kpn. A Δ*rmpD* mutant with wild-type level CPS biosynthesis but decreased HMV was shown to be more adherent to J774A.1 macrophage cells in culture and was significantly more internalized by these cells than the wild-type strain. This was further validated in a murine pneumonia model (intranasal infection), where the Δ*rmpD* mutant was readily cleared by the animals and was not detected in the lung or spleen at 72 h post-infection, compared with the wild type, highlighting a virulence and dissemination defect (26). On the other hand, a Δ*rmpC* mutant with wild-type level HMV but decreased CPS biosynthesis adhered poorly to and was less internalized by bone marrow-derived macrophages, similar to the wild type, suggesting that HMV, not the CPS, was essential for poor adhesion and resistance to phagocytosis (25). This notion received support from a later study, which showed that in the *rmpADC*-positive Kpn strain KPPR1, HMV^low^/CPS^WT^ mutants (Δ*sdhA*) adhere more to A549 cells (a human alveolar epithelial cell line), reconfirming earlier observations that HMV plays a significant role in limiting adhesion. On the other hand, an HMV^WT^/CPS^low^ mutant (Δ*pgm*) was more sensitive to the antimicrobial effects of human serum (31), suggesting that HMV prevents immune cell adhesion, while the CPS protects against complement-mediated killing. These findings garnered further support from another study that tested the binding of HMV and non-HMV Kpn isolates to lung epithelial cells, intestinal cells, and macrophages, confirming that HMV, not CPS, contributes to poor adhesion and phagocytosis (80). Taken together, these findings support a model in which HMV largely contributes to evading phagocytosis by reducing adherence to immune cells and enhancing HMV Kpn’s potential to disseminate to distal body sites (Fig. 2).

Besides inactivating mutations, promoter variations can also fine-tune the expression of the *rmpADC*/*rmpA2* loci and alter Kpn’s infection program (Fig. 2). A recent study identified five distinct variations in the promoter region upstream of the *rmpA* gene differing in the length of the poly(T) tract (81). They are named P9T, P10T, P11T, P12T, and P13T according to the number of poly(T) tracts in the promoter sequence. The authors show that P11T is the most frequently observed promoter variant and that P11T and P12T exhibit strong promoter activity. On the other hand, P9T and P10T have the weakest promoter activity, and the P13T variant has moderate promoter activity. These variations in the promoter sequences are not without reason. Weaker promoters have been shown to contribute to lower HMV, leading to better adhesion to epithelial cells in the urinary tract and driving success in this niche. For example, in a Kpn isolate carrying a P11T promoter, a single-nucleotide (T) deletion has been linked to lower *rmpA* expression, leading to greater success in the urinary tract (81). Furthermore, nearby insertion sequences can also influence the expression of the *rmpADC* locus. For example, the excision of an IS*Kpn26* element located upstream of the *rmpADC* promoter (found in ~53% of *rmpA*-positive Kpn isolates) has been shown to cause a shift from HMV to hypomucoidy (76). Thus, mutations in the coding sequence, the promoter, and upstream insertion sequences can enable subpopulations of Kpn to switch from an HMV to a non-HMV state, as may be necessary during infection (Fig. 2).

In addition to on/off states, the *rmpADC* locus is subject to complex regulation at multiple levels, allowing precise control of this virulence phenotype (Fig. 2). Promoter fusion experiments revealed that expression from the *rmpADC* promoter is decreased in the absence of the CPS biosynthesis regulators RcsB, KvrA, and KvrB (25). The Rcs phosphorelay is well known to regulate CPS biosynthesis in Kpn and colanic acid synthesis in *E. coli* (82). KvrA and KvrB belong to the MarR-family regulators. While KvrA was found to regulate both the CPS biosynthesis and the *rmpADC* loci, KvrB regulates the *rmpADC* locus (25) (Table 3). It is to be noted that we discuss only the CPS biosynthesis regulators that also co-regulate the *rmpADC* locus, and not all known regulators of CPS biosynthesis. This is important because a basal level of CPS biosynthesis is necessary for HMV manifestation, and CPS levels may indirectly affect HMV. There are excellent, recent review articles that the reader may refer to for a detailed account of the regulation of CPS biosynthesis in Kpn (32, 36, 83). RNA interaction profiling by ligation and sequencing (RIL-seq) identified that several genes in the CPS biosynthesis locus were targets for small-RNA (sRNA)-mediated regulation (84). The sRNA RybB was identified to interact with the *rmpA* transcript. In addition, OmrB was identified as interacting with transcripts of *rmpA* and *kvrA*. However, overexpression of RybB did not affect CPS or HMV levels, whereas overexpression of OmrB decreased both CPS and HMV levels. The authors showed that OmrB base-pairs with the *kvrA* transcript, exerting regulatory control over CPS biosynthesis and, by extension, over HMV in Kpn SGH10 (84) (Table 3). In Kpn ATCC 43816, a similar RIL-seq study identified that the sRNA ArcZ interacts with several CPS biosynthesis and HMV-related genes (85). The study showed that overexpression of ArcZ repressed HMV by directly interacting with the *mlaA* transcript. The loss of *mlaA* leads to decreased HMV and attenuation of virulence in a murine pneumonia model. MlaA, along with MlaBCDEF, is involved in the phospholipid retrograde transport mechanism that maintains lipid asymmetry in the outer membrane. By systematically altering active-site residues required for phospholipid binding, the authors propose a model wherein phospholipid retrograde transport is crucial for HMV. Taken together, these studies highlight how CPS biosynthesis and *rmpADC*-mediated HMV may be coregulated (Table 3). The ferric uptake regulator (Fur) also coregulates the CPS and *rmpADC* loci in response to iron levels. A Fur-binding site was identified within the *rmpADC* promoter region (77), and the loss of *fur* has been shown to derepress expression of the *rmpADC* locus and result in persistent HMV that is insensitive to iron availability (86). Thus, HMV is also positively influenced by iron availability in the infection niches and by Kpn’s ability to scavenge the limiting nutrient from the host using specialized siderophore systems. The availability of other nutrients, such as amino acids and sugars, can also modulate HMV in Kpn (Fig. 2). The amino acid arginine, which is detected in the respiratory tract during infection, has been shown to enhance the HMV of *rmpADC*-positive Kpn (87). This effect depends on the ArgR regulator, which activates transcription from the *rmpADC* locus in the presence of arginine, without affecting the expression of CPS biosynthesis genes. A highly conserved ArgR-binding box was identified in the *rmpADC* promoter (Fig. 2). The same group has also shown that the import, not the catabolism, of sugars (D-galactose, D-glucose, D-mannose, L-rhamnose, L-arabinose, L-fucose, L-xylose, and N-acetyl-D-glucosamine) can suppress HMV (88). This effect appears to be due to downregulation of the *rmpADC* locus and is independent of CPS biosynthesis (Fig. 2). Interestingly, a transposon screen for mutants relieved of sugar-suppressed HMV identified various genes belonging to functional categories such as carbon-responsive global regulation (*cyaA*, *csrD*, and *crp*), nitrogen regulation (*ntrBC*, *ptsN*, and *rpoN*), and peptide transport (*sapA* and *sapB*), among others. Notably, *rmpADC*-positive Kpn cells exposed to sugars were more adherent to epithelial cells and associated better with porcine gastric mucin, suggesting that the abundance of these sugars in the infection niches of Kpn could also influence HMV.

**TABLE 3:** The HMV phenotype is modulated by environmental conditions through regulators, cellular metabolism, and CPS modifications

Category	Factor	Effect on HMV	Mechanism of action	Reference
Regulators	RmpA	Increases	Autoactivation of the *rmpADC* locus	(21)
	RcsB	Increases	Activation of CPS biosynthesis locus and *rmpADC*	(25)
	KvrA	Increases	Activation of CPS biosynthesis locus and *rmpADC*	(25)
	KvrB	Increases	Activation of the *rmpADC* locus	(25)
	OmrB (sRNA)	Decreases	Base-pairing with the *kvrA* transcript	(84)
	ArcZ (sRNA)	Decreases	Base-pairing with the *mlaA* transcript.	(85)
	FNR	Increases	Unknown, but independent of *rmpADC*/*rmpA2* loci.	(89)
	OmpR	Increases	Unknown.	(90)
	ArgR	Increases	Activation of the *rmpADC* locus in the presence of arginine	(87)
	Fur	Decreases	Fur represses the *rmpADC* locus	(86)
Environmental conditions	Temperature: 37°C	Increases compared to 20°C–25°C	Unknown. In *rmpADC* (+) ve strains.	(91)
	Temperature: 20°C–25°C	Increases compared to 37°C	Unknown. In *rmpADC* (−) ve strains.	(91)
	Acidic pH	Modestly decreases	Unknown. In *rmpADC* (+)ve strains only.	(63)
	Alkaline pH	Modestly increases	Unknown. In *rmpADC* (+)ve strains only.	(63)
	Sugar import	Decreases	Repression of the *rmpADC* locus	(88)
	Human urine	Decreases	Unknown	(63)
	Co-culture with Acanthamoebae	Increases	Unknown	(92)
Resistance to antibiotics	Tigecycline	Decreases	Unknown	(93)
	Colistin	Decreases	Unknown	(94)
	Ceftazidime-avibactam	Decreases	Unknown	(95)
Metabolism-related genes	AceE	Increases	Unknown. E1 component of pyruvate dehydrogenase.	(31)
	SdhA	Increases	Unknown. Flavoprotein component of succinate dehydrogenase	(31)
Capsule modifications	WcsU	Increases	O-acetylation of the CPS	(96)
	KpACE	Decreases	Deacetylation of the CPS	(96)
	Sialic acid	Increases	Chemical modification of the CPS with sialic acid enhances HMV	(34)

Apart from nutrients available in the infection niches, prevailing environmental conditions, such as temperature, pH, and oxygen availability, can also modulate HMV (Table 3). Interestingly, the effect of temperature on HMV appears to be dependent on how HMV is manifested, i.e., through the *rmpADC*/*rmpA2* loci or other mechanisms (91). A genomic epidemiological study of 156 HMV Kpn isolates reported that *rmpADC*/*rmpA2* loci-bearing HMV strains produced longer strings at 37°C compared to those at room temperature (20°C–25°C), indicating enhanced HMV at 37°C. On the other hand, the HMV strains, which do not carry *rmpADC*/*rmpA2* loci, produced longer strings and enhanced HMV at room temperature compared to 37°C. The mechanism underlying this phenomenon is not fully understood, and the observations may also be influenced by the effects of incubation temperature on CPS biosynthesis. In addition to temperature, microaerobic conditions also influenced HMV by downregulating the expression of both CPS biosynthesis and *rmp*-locus genes, leading to decreased HMV (97). However, overexpression of *rmpD* in the wild-type strain, even under microaerobic conditions, restored HMV. This finding is significant as it may allow Kpn to alter its infection program in microaerobic niches such as the gastrointestinal tract, wounds, and deep tissues, where HMV may be disadvantageous due to a greater need for adhesion and invasion. Similarly, unknown factors in human urine were found to suppress *rmpD*-mediated HMV without affecting CPS biosynthesis (Fig. 2). Initially, the authors suspected that urine pH might influence HMV. They showed that growth in Luria Bertani (LB) medium at an alkaline pH of 9 modestly increased HMV (sedimentation resistance), while growth at an acidic pH of 5 modestly decreased HMV (63). However, this decrease was not as pronounced as that observed during Kpn growth in urine. Although the factor suppressing HMV was elusive, the authors demonstrated that expression from the *rmp* locus was significantly decreased, while expression of CPS biosynthesis genes remained unaffected. This suggests that Kpn can sense the urinary tract environment via this mechanism, decrease its HMV, adhere more to bladder epithelial cells, and successfully cause a urinary tract infection.

Kpn is exposed to antibiotics during treatment after an infection. Antibiotic exposure and the emergence of antibiotic resistance in Kpn can modulate HMV. Sub-minimum inhibitory concentration of levofloxacin led to a decrease in the expression of *rmpA* and a reduction in both CPS biosynthesis and HMV (98). Similarly, at sub-minimum inhibitory concentrations, rifampicin decreased HMV by downregulating *rmpA* (99) (Fig. 2). However, the mechanism by which *rmpA* gene expression is downregulated by rifampicin remains unclear. Rifampicin is known to act by binding the RpoB subunit of RNA polymerase and inhibiting translation. However, resistance can emerge through target-modifying mutations in the *rpoB* gene. A later study by the same group showed that rifampicin-resistant mutants bearing *rpoB* substitutions were insensitive to rifampicin’s HMV-suppressive effects (100). These studies suggest that antimicrobials, and possibly other small molecules, could have HMV-suppressive effects and could be repurposed as anti-virulence agents. However, given the diverse niches Kpn infects and the distinct infection programs that it operates, such anti-virulence therapies will have to be designed niche-specifically. The emergence of antimicrobial resistance in Kpn is also known to affect its fitness and virulence. Experimentally evolved tigecycline-resistant mutants (93), colistin-resistant (94), and ceftazidime-avibactam-resistant mutants of Kpn (95) had decreased HMV compared to the parent strains (Table 3). The cost of acquiring antibiotic resistance may sometimes need to be offset by a compromise in virulence. However, it was also shown that antibiotic-resistant variants could further evolve to achieve fitness levels comparable to that of the parent via compensatory variations (95).

Several studies have identified roles for other modulators of the HMV phenotype, although the mechanisms by which they act remain unclear (Table 3). In the hypervirulent and hypermucoviscous Kpn strain NTUH-K2044, carrying both *rmpADC* and the *rmpA2* loci, the inactivation of the fumarate and nitrate reduction regulator (FNR) was shown to abolish HMV, suggestive of its positive regulatory role in HMV manifestation (89). FNR does not appear to modulate HMV by regulating the expression of the *rmpADC*/*rmpA2* loci. This finding raises the possibility of additional FNR-regulated loci contributing to HMV in Kpn. Similarly, the loss of the osmotic stress response regulator OmpR was found to decrease CPS biosynthesis modestly and HMV in a transposon screen in the strain KPPR1 (31). However, in ATCC 43816, the parent strain of KPPR1, CPS biosynthesis is modestly, but not significantly decreased, while HMV is significantly decreased upon loss of OmpR. The authors note that the decrease in HMV is not due to reduced expression of the *rmpADC* locus, as its expression remains at wild-type levels in the Δ*ompR* mutant. They then show that OmpR binds the promoter of the F-type ATP synthase operon and represses gene expression, arguing that elevated cellular ATP levels may negatively impact HMV through an unknown mechanism (90). The regulation of HMV by OmpR is particularly significant because OmpR acts as a critical sensor for environmental osmolarity and acid stress. It is well documented in the literature that fluctuations in urinary tract osmolarity can modulate bacterial virulence (101, 102). Therefore, osmolarity-driven modulation of OmpR may serve as a mechanism to regulate HMV in this specific niche, ultimately influencing the outcome of the infection.

Other genes with cellular metabolism-related functions have also been shown to modulate HMV (Table 3). For example, in a transposon screen of the strain KPPR1, mutants with HMV^low^/CPS^high^ (Δ*aceE*) and HMV^low^/CPS^WT^ (Δ*sdhA*) were identified (31). The gene *aceE* encodes the E1 component of pyruvate dehydrogenase, while *sdhA* encodes the flavoprotein component of succinate dehydrogenase, both of which participate in metabolism. However, how exactly their loss decreases HMV is not mechanistically understood. Finally, the CPS modification also enhanced HMV in the *rmpADC*-positive isolate ATCC 43816. A pair of loci encoding an acetylesterase (KpACE) and a putative acetyltransferase (WcsU) was identified to control O-acetylation of the CPS, thereby modulating HMV (96). WcsU appears to acetylate the CPS, while KpACE deacetylates it, resulting in the reduction of HMV when KpACE is overexpressed or in a Δ*wcsU* mutant. It is important to note that HMV is merely reduced rather than abolished upon deletion of *wcsU*. The ability to alter infection programs by modulating HMV is not limited to hypervirulent strains bearing *rmpADC*/*rmpA2*. Even classical Kpn isolates can readily alter their infection programs through mutational events in the capsule biosynthesis and export machinery, particularly in the capsule locus-associated tyrosine autokinase gene *wzc*. For example, in ST258 isolates, which are considered classical and not highly virulent, the emergence of mutations in *wzc* resulting in different Wzc variants (G565A/S/R and A535E) was shown to confer phagocytosis resistance (J774 murine macrophage cell line) and enhanced virulence in a murine UTI model. Similarly, a strain bearing the Wzc variant L74P, obtained from a patient suffering from UTI, was highly lethal in three different (UTI, intraperitoneal, and bloodstream) murine models of infection and caused pyogenic liver abscesses in infected animals (53), suggestive of enhanced ability to disseminate within the host. The authors proposed a model where classical Kpn isolates with normal CPS can colonize the bladder, form biofilms on catheters, and can also invade the bladder epithelial cells, possibly due to better adherence to biotic and abiotic surfaces. In contrast, Kpn variants carrying *wzc* mutations were resistant to phagocytosis, could disseminate effectively to distal body sites, but were not adept at forming biofilms or invading bladder cells. Other studies reporting the emergence of HMV isolates bearing Wzc variants also indicate enhanced resistance to phagocytosis due to reduced adhesion to epithelial and immune cells and increased dissemination potential (Table 2). In the study by Khadka and colleagues (63), although *rmpD*-mediated HMV was suppressed by human urine in an *rmpADC*-positive Kpn strain, Kpn could still alter its infection program to become HMV via *wzc* mutations. Surprisingly, the HMV phenotype caused by *wzc* mutations was insensitive to the HMV-suppressing effects of human urine. Enhanced phagocytosis resistance can be beneficial in other environments as well. For example, Huang and colleagues (92) found that non-HMV Kpn clinical isolates cocultured with *Acanthamoeba* sp., a phagocytic predator of bacteria, increased CPS levels, thickness, and HMV (Table 3). Although the study did not screen for *wzc* variations in the bacteria that evolved HMV, *wzc* mutations that decrease adhesion and enhance phagocytosis resistance likely occurred and were beneficial during coculture. These studies highlight how Kpn can alter its infection program by acquiring a single mutation in *wzc*. Although it may appear that this switch to the HMV state is one-way, there are likely variants with *wzc* mutations at low frequency that may or may not be selected depending on the niche and prevailing conditions. Given the current focus on *rmpADC*/*rmpA2*-mediated HMV in Kpn, the prevalence of HMV-conferring *wzc* variants at the population level remains unknown and warrants large-scale studies.

## CONCLUSION

The study of HMV in Kpn has evolved from a simple observation during routine diagnosis into a complex field revealing how bacteria fine-tune their physical properties for survival. Recent research has provided a critical paradigm shift, demonstrating that HMV is a trait characterized by increased chain length and uniformity in the CPS chains. The key player in the manifestation of HMV appears to be Wzc, the tyrosine autokinase encoded by the CPS biosynthesis locus. In *rmpADC*/*rmpA2*-positive strains, RmpD is known to interact with Wzc. How RmpD modulates Wzc activity to enhance CPS chain length and uniformity remains to be understood. In particular, the conformational changes introduced in Wzc upon binding by RmpD may help explain how RmpD enhances the chain length and uniformity of the Kpn CPS. It is also unclear why the prevalence of *rmpADC* and *rmpA2* loci at the population level is low (~7%) if they confer an advantage during infection, especially for dissemination. Perhaps there are fitness costs associated with the maintenance of large virulence plasmids and the manifestation of RmpD-mediated HMV. The discovery that classical multidrug-resistant strains can acquire HMV through spontaneous missense mutations in *wzc* is of profound clinical concern. These mutations allow non-hypervirulent lineages to bypass the need to acquire large virulence plasmids to manifest HMV. Though several variants of Wzc, conferring HMV, have been reported, how these mutations affect Wzc structure, Wzc phosphorylation status, CPS chain length, CPS chain length uniformity, CPS spatial arrangement, and consequently HMV remains unknown. Since Wzc in its dephosphorylated state is believed to assemble with Wzy into the polymerization platform (40), it is tempting to speculate that the duration for which the polymerization platform stays intact may determine the chain length of the synthesized polymer. More regular cycling between assembly and disassembly of the polymerization platform (Wzc and Wzy), along with longer durations in the assembled state (dephosphorylated Wzc), may cause longer and more uniform CPS chain lengths, driving HMV. In contrast, irregular cycling between assembly and disassembly of the polymerization platform, driven by more random Wzc dephosphorylation and phosphorylation events, may lead to the production of non-uniform CPS chains as observed in non-HMV strains. Along similar lines, it is interesting to speculate on the role of *wzb*, which encodes the phosphatase that dephosphorylates Wzc in HMV. Given the importance of Wzc phosphorylation status in HMV manifestation, it is surprising that mutations in *wzb* or its promoter, as well as novel proteins that may modulate Wzb activity, have not yet been reported. These possibilities cannot be ruled out and warrant further study.

Worryingly, evidence suggests that antibiotic stress, particularly from treatments such as ceftazidime-avibactam, can induce these HMV-conferring mutations, effectively turning a standard infection into an invasive one during the course of therapy. It is also unclear if other antibiotics have similar effects on HMV emergence. Another important concern stemming from this knowledge is the lack of large-scale studies on the population-level prevalence of HMV-conferring *wzc* mutations in Kpn. Arguably, this is also difficult to address, as only variants that get selected in an infection niche will be isolated and sequenced. Analysis of metagenomic next-generation sequencing data from patient-derived samples across different infection niches may shed more light on this. Although HMV alone may not confer hypervirulence (43), when combined with other hypervirulence markers, such as aerobactin, it can certainly enhance the virulence of Kpn. In countries like India, where the SL231 lineage carrying the hypervirulence-associated siderophore aerobactin biosynthesis locus is prevalent (37), the *wzc*-mutation-mediated emergence of HMV is even more concerning and warrants further study.

Beyond its genetic origins, the regulation of HMV reveals an intricate infection program adapted to specific host niches. Kpn does not maintain HMV and may not need it at all times. Instead, it uses sophisticated regulatory networks modulated by environmental cues to toggle the phenotype. Some studies report anti-virulence agents capable of decreasing the expression of *rmpD*-mediated HMV and/or CPS biosynthesis in Kpn. However, the applicability of these agents against Kpn, which has an open pan-genome and often strain-dependent regulatory networks, needs further investigation. In conclusion, recent findings on HMV in Kpn have answered several key questions on how the phenotype manifests and is regulated. However, they have also raised many more important questions, and addressing these gaps will be critical to tackling the global threat posed by HMV and hypervirulent Kpn lineages.
